# Complete mitochondrial genome of the smallmouth hardyhead (*Atherinosoma microstoma*) and its phylogenetic position among the Atheriniform fishes

**DOI:** 10.1080/23802359.2017.1334524

**Published:** 2017-05-28

**Authors:** Davin H. E. Setiamarga

**Affiliations:** a Department of Applied Chemistry and Biochemistry, National Institute of Technology, Wakayama College, Gobo City, Wakayama, Japan;; b The University Museum, The University of Tokyo, Tokyo, Japan

**Keywords:** Atheriniformes, mitogenome, percomorph teleosts, small-mouth hardyhead

## Abstract

In this paper, I report the full mitochondrial genome sequence of the smallmouth hardyhead (*Atherinosoma microstoma*), an endemic marine fish from the shallow coastal waters of southeastern Australia. The mitogenome is 16,573 bp-long with the standard 37 genes all included, with a genomic structure typical of a vertebrate mitogenome. In order to confirm the phylogenetic position of this species, phylogenetic trees were inferred using a data set including publicly available 28 atherinomorph, nine percomorph, and two outgroup mitogenome sequences. The complete mitogenome data of *A. microstoma* reported here will be useful for further genetics, phylogeography, and phylogenetics studies involving this species.

The smallmouth hardyhead, *Atherinosoma microstoma*, is one of the only two known species of its genus. Taxonomically, it belongs to the family Atherinidae, order Atheriniformes, series Atherinomorpha. The species is native to Australia, and distributed along the shallow coastal waters of southeastern Australia. Here, we report the full mitochondrial genome (mitogenome) sequence of this species. Tissue sample was collected from an individual specimen from the Fish Collection of the Ichthyology Department of the Australian Museum (Voucher No. I.40457-001). Detailed information of the specimen can be obtained online (http://collections.australianmuseum.net.au/amweb/pages/am/Display.php?irn=8142666&QueryPage=%2Famweb%2Fpages%2Fam%2FAdvQuery.php&highlight_term=). PCR-based mitogenome sequencing using fish versatile primers was conducted in accordance to what was reported previously (Miya & Nishida 1999; Setiamarga et al. 2008). Assembled mitogenome sequence was annotated using the MitoAnnotator on the MitoFish homepage (http://mitofish.aori.u-tokyo.ac.jp/annotation/input.html).

The newly sequenced mitogenome of *Atherinosoma microstoma* was 16,573 bp long (Registered to DDBJ). Its genomic structure is similar to a typical vertebrate/euteleost mitogenome with some interesting exceptions. (1) There are 13 protein-coding, two rRNA, and 22 tRNA genes, (2) There are two control regions, located between the tRNA-Pro and tRNA-Phe (873 bp-long) and the ND6 and ND5 genes (213 bp-long); (2) Most genes are coded on the H chain, except for ND6 and eight tRNA genes (tRNAPro, tRNAGln, tRNAAla, tRNAAsn, tRNACys, tRNATyr, tRNASer(UCN), and tRNAGlu)); (3) ND4L is located on the H chain, unlike a that of a typical vertebrate. The total GC content the mitogenome was 46.4%.

In order to check the phylogenetic position of *A. microstoma* molecularly, we collected publicly available mitogenomes of 12 Atheriniformes, eight Beloniformes, eight Cyprinodontiformes, nine non-atherinomorph percomorphs, and two non-percomorph outgroups from GenBank, and then built a data set for phylogenetic analyses. I conducted a maximum likelihood phylogenetic analysis, which methods are detailed in the legend of Figure 1. The resulting phylogeny was congruent with previous mitogenome (e.g. Kawahara et al. 2008; Setiamarga et al. 2008, 2009) and nuclear gene marker (e.g. Betancur et al. 2013) studies. The monophylies of the order Atheriniformes, superorder Cyprinodontea, and series Atherinomorpha are well supported. The monophylies of the two suborders of Atheriniformes, Atherinopsoidei and Atherinoidei were supported with *A. microstoma* included in the latter suborder.

**Figure 1.**
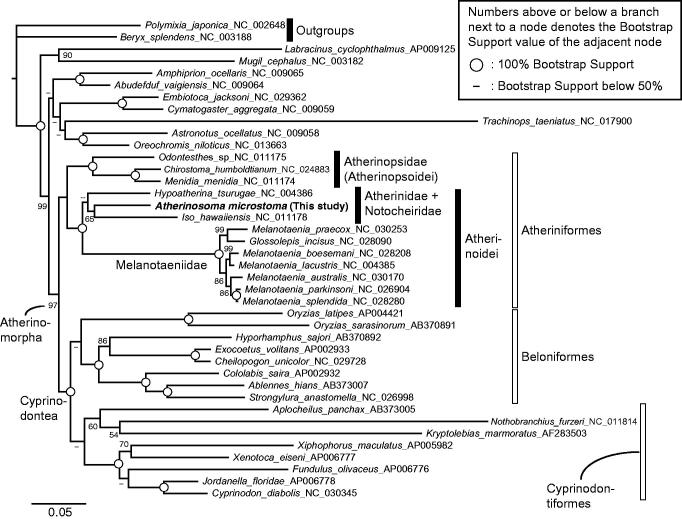
A maximum likelihood (ML) molecular phylogenetic tree, inferred using the program RAxML (Stamatakis 2014). Phylogenetic analyses were conducted on a data matrix (11,082 positions) including all the concatenated nucleotide sequences of the mitogenomes except the third codon positions. The ND6 gene was excluded from the analysis. Gene sequences were aligned individually using the online version of MAFFT under default settings (http://mafft.cbrc.jp/alignment/server/; Katoh & Standley 2013). Aligned sequences were individually edited using the online version of GBlocks using the least stringent settings (http://molevol.cmima.csic.es/castresana/Gblocks_server.html; Castresana, 2000). Data partitions were determined using the program PartitionFinder ver. 2 (Lanfear et al. 2017). Partitioned ML analyses were performed with RAxML-GUI ver. 1-5b1 (Silvestro & Michalak 2012), with the GTR + Γ + I nucleotide substitution model (Yang 1994). The rapid bootstrap analyses were conducted with 1000 replications, with four threads running in parallel.

The result presented here will be useful for future phylogeography and population genetics studies of this Australian endemic species. Meanwhile, the addition of the full mitogenome sequence data of this species will be useful for future molecular phylogenetics studies of the Atheriniformes, Atherinomorpha, and Percomorpha in general.
